# A quick guide to survey research

**DOI:** 10.1308/003588414X13824511649454

**Published:** 2014-01

**Authors:** A Misro, M Hussain, TL Jones, MA Baxter, V Khanduja

**Affiliations:** Ealing Hospital NHS Trust,UK

We read the article on survey research with interest and would like to make the following comments:

First, there is no mention of multimodal surveys and the expected response rate in this article. Multimodal surveys are carried out to improve the response rate. This is due to the fact that when an individual does not respond to email reminders, there is a high likelihood of issues such as spam filtering or that non-responders may not be frequent users of the internet. These people can be reached through alternative means like postal questionnaires or telephone surveys. To our knowledge, email surveys without a follow-up email can generate response rates up to 30%.

Second, email personalisation, reminders and incentives are some of the important tools that can improve response rates.

Third, we would like to reiterate the importance of pilot surveys. A job well begun is a job half done. The importance of this crucial step cannot be overemphasised. Pilot surveys should be circulated among a small group that includes individuals who represent all the subgroups (eg junior doctors, senior doctors, managers, consultants, students). The main objective of the pilot is to obtain user feedback with a view to modifying the questionnaire before embarking on the main project.

Finally, we differ from the authors’ opinion regarding survey expenses. Surveys can be carried out by inexpensive means. Nevertheless, they can be quite time consuming, especially with delayed responses.

